# Blood lactate normalization following venoarterial ECMO institution for refractory cardiogenic shock

**DOI:** 10.1186/cc14226

**Published:** 2015-03-16

**Authors:** M Bottiroli, D Decaria, T Maraffi, S Nonini, F Milazzo, R Paino

**Affiliations:** 1 Anestesia Rianimazione 3, A.O. Niguarda, Milan, Italy

## Introduction

Venoarterial (VA) ECMO is used to support patients with refractory cardiogenic shock (CS). Elevated lactate level (>2 mmol/l) is an indicator of end-organ hypoperfusion. We hypothesize that the lactate (LAC) normalization had prognostic value in this cohort of patients.

## Methods

We performed a retrospective observational study on patients admitted to the ICU for refractory CS from January 2010 to November 2014. Patients with postcardiotomy and/or post-transplant CS were excluded. Demographics, clinical, hemodynamic and biochemical values were collected. LAC was measured on arterial blood before ECMO institution (LAC0) and after 48 hours (LAC48). Lactate clearance was calculated as follows: (LAC0 - LAC48) / LAC0 × 100. Data were analyzed by comparative statistic; sensibility and specificity were tested with ROC.

## Results

**Figure 1:**
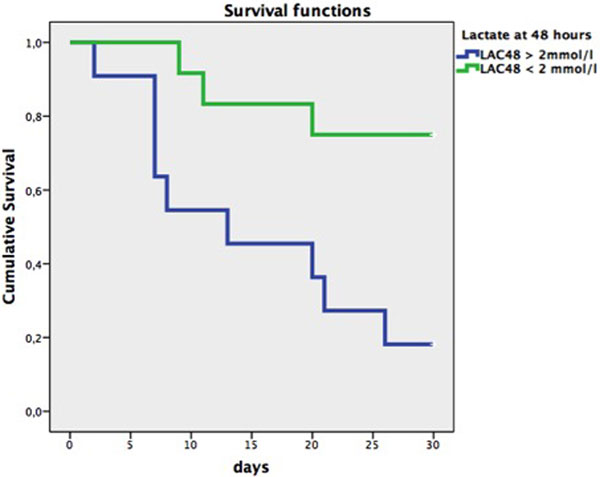


Twenty-three patients underwent VA ECMO for refractory CS in the study period. Etiologies of CS were: 11 acute myocarditis, five acute myocardial infarction and seven acute decompensation of chronic cardiomyopathy. The median time of ECMO was 10 days (4 to 15). Thirteen patients died during hospital stay and 10 survived. Three patients were bridged to LVAD and two to heart transplant; eight were bridged to recovery. The main cause of ICU death was multiple organ dysfunction (12/13). Nonsurvivors showed significantly higher LAC0 (5 (2 to 6) vs. 8 (5 to 11), *P *= 0.021). Lactate clearance at 48 hours was not significantly different between survivors and nonsurvivors (79%, 95% CI = 67 to 86 vs. 60%, 95% CI = 32 to 72, *P *= 0.08). However, LAC48 was predictive for ICU mortality (AUC 0.82; 95% CI = 0.64 to 1.0; *P *= 0.011). ROC curve analysis identified the accuracy was highest by setting the lactate <2 mmol/l. Patients that did not normalize lactate (LAC <2 mmol/l) after 48 hours despite hemodynamic restoration had poorer outcome at 30 days, as is shown in the Kaplan-Meier curve (logrank *P *= 0.006) (Figure 1).

## Conclusion

Failing to normalize patient's LAC in the first 48 hours of VA ECMO assistance for CS is a predictor of ICU mortality. Targeting LAC level <2 mmol/l at 48 hours post ECMO institution might be a reasonable goal for these patients.

